# Effects of different serum conditions on osteogenic differentiation of human adipose stem cells *in vitro*

**DOI:** 10.1186/scrt165

**Published:** 2013-02-15

**Authors:** Laura Kyllönen, Suvi Haimi, Bettina Mannerström, Heini Huhtala, Kristiina M Rajala, Heli Skottman, George K Sándor, Susanna Miettinen

**Affiliations:** 1 Adult Stem Cells Group, Institute of Biomedical Technology, University of Tampere, Biokatu 8, Tampere FI-33014, Finland; 2 BioMediTech, Biokatu 10, Tampere FI-33520, Finland; 3 Science Center, Tampere University Hospital, Biokatu 10, Tampere FI-33521, Finland; 4 Department of Biomaterials Science and Technology, University of Twente, De Horst 2, Enchede 7522 LW, The Netherlands; 5 Tampere School of Public Health, University of Tampere, Medisiinarinkatu 3, Tampere FI-33014, Finland; 6 Cardiology Group, Institute of Biomedical Technology, University of Tampere, Biokatu 8, Tampere FI-33014, Finland; 7 Ophthalmology Group, Institute of Biomedical Technology, University of Tampere, Biokatu 8, Tampere FI-33014, Finland; 8 Department of Oral and Maxillofacial Surgery, Institute of Dentistry, University of Oulu, Aapistie 3, Oulu FI-90014, Finland

## Abstract

**Introduction:**

Currently, human adipose stem cells (hASCs) are differentiated towards osteogenic lineages using culture medium supplemented with L-ascorbic acid 2-phosphate (AsA2-P), dexamethasone (Dex) and beta-glycerophosphate (β-GP). Because this osteogenic medium (OM1) was initially generated for the differentiation of bone marrow-derived mesenchymal stem cells, the component concentrations may not be optimal for the differentiation of hASCs. After preliminary screening, two efficient osteogenic media (OM2 and OM3) were chosen to be compared with the commonly used osteogenic medium (OM1). To further develop the culture conditions towards clinical usage, the osteo-inductive efficiencies of OM1, OM2 and OM3 were compared using human serum (HS)-based medium and a defined, xeno-free medium (RegES), with fetal bovine serum (FBS)-based medium serving as a control.

**Methods:**

To compare the osteo-inductive efficiency of OM1, OM2 and OM3 in FBS-, HS- and RegES-based medium, the osteogenic differentiation was assessed by alkaline phosphatase (ALP) activity, mineralization, and expression of osteogenic marker genes (runx2A, DLX5, collagen type I, osteocalcin, and ALP).

**Results:**

In HS-based medium, the ALP activity increased significantly by OM3, and mineralization was enhanced by both OM2 and OM3, which have high AsA2-P and low Dex concentrations. ALP activity and mineralization of hASCs was the weakest in FBS-based medium, with no significant differences between the OM compositions due to donor variation. However, the qRT-PCR data demonstrated significant upregulation of runx2A mRNA under osteogenic differentiation in FBS- and HS-based medium, particularly by OM3 under FBS conditions. Further, the expression of DLX5 was greatly stimulated by OM1 to 3 on day 7 when compared to control. The regulation of collagen type I, ALP, and osteocalcin mRNA was modest under induction by OM1 to 3. The RegES medium was found to support the proliferation and osteogenic differentiation of hASCs, but the composition of the RegES medium hindered the comparison of OM1, OM2 and OM3.

**Conclusions:**

Serum conditions affect hASC proliferation and differentiation significantly. The ALP activity and mineralization was the weakest in FBS-based medium, although osteogenic markers were upregulated on mRNA level. When comparing the OM composition, the commonly used OM1 was least effective. Accordingly, higher concentration of AsA2-P and lower concentration of Dex, as in OM2 and OM3, should be used for the osteogenic differentiation of hASCs *in vitro*.

## Introduction

The osteogenic potential of human adipose stem cells (hASCs) has recently stimulated interest in clinical bone tissue engineering [1,2]. This multipotent population of cells, isolated from the stromal vascular compartment of adipose tissue, was originally characterized by Zuk and co-workers [3]. It was soon discovered that hASCs are able to differentiate toward osteogenic, adipogenic, myogenic, and chondrogenic lineages *in vitro*, when treated with appropriate inducing factors [3]. Since their discovery, different approaches have been developed to enhance osteogenic capacity of hASCs. Much of the research has concentrated on the osteo-induction of hASCs via growth factors such as bone morphogenetic proteins (BMPs) [4-6]. Although considerable research has been devoted to BMPs, their cost-effectiveness and safety in clinical use have been under controversy [7-10]. Recent studies have also questioned whether hASCs are responsive to BMPs at all [11]. Therefore, efficient methods for osteo-induction of hASCs are still required.

Commonly, osteogenic differentiation of mesenchymal stem cells (MSCs) in *in vitro *culture has been managed by supplementing the growth medium with 50 µM L-ascorbic acid 2-phosphate (AsA2-P), 100 nM dexamethasone (Dex) and 10 mM beta-glycerophosphate (β-GP) [11-14]. Because this osteogenic medium (OM) was initially generated for the differentiation of bone marrow-derived mesenchymal stem cells (BMSCs) [15], the component concentrations may not be optimal for the differentiation of hASCs [16]. Although BMSCs and ASCs possess many similar characteristics, their response to inductive stimuli may not be identical [12,17,18]. Whereas the combined and separate effects of Dex and AsA2-P have been largely studied with BMSCs [15,19-21], there are only limited studies with hASCs [16,22]. For example, de Girolamo and co-workers suggested that OM with lower Dex and higher AsA2-P concentration could be more effective with hASCs than the commonly used OM [16]. However, this study was conducted using two donor cell lines and more importantly only fetal bovine serum (FBS)-containing medium. Therefore, we conducted a preliminary screening using different osteogenic supplement concentrations in human serum (HS)-based medium. Based on these preliminary results, two efficient compositions (OM2 and OM3) were chosen for further comparison in different serum conditions with the commonly used OM (referred here as OM1).

So far, the effects of OM on hASC differentiation have been defined mostly using FBS-containing medium [12,13,16]. While FBS-based medium may be acceptable for the *in vitro *experiments, exposure to undefined animal-derived products poses a risk in clinical stem cell therapies [23,24]. Replacing animal-derived products such as FBS with human serum, human platelet lysate, platelet-rich plasma or defined xeno-free alternative, significantly enhances the safety and quality of stem cells for therapeutic approaches [23,25]. In addition to safety issues, serum conditions can have a significant effect on stem cell characteristics such as differentiation capacity and proliferation [26,27]. In order to define how serum conditions affect the osteogenic differentiation of hASCs, we tested HS-based medium and a defined, xeno-free medium (RegES) in comparison to FBS-based medium to evaluate the efficiency of three different OM compositions. The fully defined xeno-free medium formulation RegES was previously developed in our institute for stem cell culture [28].

In order to produce clinically relevant *in vitro *data the effects of three different OM compositions on hASCs were compared in the present study using FBS, HS and xeno-free RegES medium.

## Materials and methods

### Ethics statement

This study was conducted in accordance with the Ethics Committee of the Pirkanmaa Hospital District, Tampere, Finland (R03058), to obtain adipose tissue samples for research purposes. The hASCs were isolated from adipose tissue samples acquired from surgeries performed in the Department of Plastic Surgery, Tampere University Hospital. The subcutaneous adipose tissue samples harvested from either abdomen or breast were obtained with a written informed consent from six female donors (mean age 53 ± 16).

### Isolation and cell culture

Human ASCs were isolated from adipose tissue samples by mechanical and enzymatic method as described previously [26,29]. Briefly, the adipose tissue samples were minced into smaller pieces and digested with collagenase type I (1.5 mg/ml; Invitrogen, Carlsbad, CA, USA), followed by centrifugation and filtering steps. The isolated hASCs were maintained and expanded in maintenance medium (MM) consisting of Dulbecco's modified Eagle's medium/Ham's Nutrient Mixture F-12 (DMEM/F-12 1:1; Invitrogen) supplemented with 1% L-alanyl-L-glutamine (GlutaMAX; Invitrogen), 1% antibiotics (100 U/ml penicillin, 0.1 mg/ml streptomycin; Invitrogen), and either 10% allogeneic HS (PAA Laboratories GmbH, Pasching, Austria) (referred as HS MM) or 10% FBS Gold (PAA Laboratories) (referred as FBS MM). FBS Gold was used for the experiments, because it has been reported to eliminate the need for extensive and time-consuming batch testing due to its constant quality. The hASCs from each donor were isolated into both HS and FBS maintenance medium. The hASCs isolated and expanded in HS medium were detached using TrypLE Select (Life Technologies, Carlsbad, CA, USA), whereas hASCs in FBS medium were detached using 1% trypsin (Lonza Biowhittaker, Verviers, Belgium). For xeno-free conditions, hASCs were first isolated and expanded in HS MM, and xeno-free RegES medium was added after hASCs were seeded onto well plates. The composition of RegES maintenance medium (RegES MM) is shown in Additional file 1. A CELLstart (Invitrogen) pre-coating of polystyrene well plates was required for the cell attachment under RegES conditions. The experiments were carried out at passages three to four.

### Characterization of the cells

In order to verify the mesenchymal origin, hASCs must meet several criteria defined by the International Society for Cellular Therapy [30]. The mesenchymal origin of hASCs used in this study was confirmed by their adherence to plastic, differentiation capacity to osteogenic, chondrogenic and adipogenic lineages *in vitro*, and by their surface marker expression.

### Flow cytometric surface marker expression analysis

Flow cytometry was used to characterize the surface marker expression of hASCs cultured in FBS and HS MM. Flow cytometric characterization comparing the surface marker expression of hASCs cultured in HS medium and xeno-free RegES medium has been reported previously [28]. Briefly, hASCs cultured in FBS and HS MM were analyzed by a fluorescence-activated cell sorter (FACSAria; BD Biosciences, Erembodegem, Belgium) as described by Lindroos and co-workers [26]. Monoclonal antibodies against CD14-PE, CD19-PE, CD49d-PE, CD73-PE, CD90-APC, CD106-PE, (BD Biosciences); CD45-FITC (Miltenyi Biotech, Bergisch Gladbach, Germany); CD34-APC, HLA-ABC-PE, HLA-DR-PE (Immunotools GmbH Friesoythe, Germany); and CD105-PE (R&D Systems Inc, Minneapolis, MN, USA) were used. Analysis was performed on 10,000 cells per sample and unstained cell samples were used to compensate for the background autofluorescence levels.

### Analysis of multipotent differentiation capacity

The multipotent differentiation of hASCs was conducted in RegES medium supplemented with corresponding adipogenic, osteogenic and chondrogenic components. The multipotent differentiation capacity of hASCs cultured in FBS and HS medium has been shown previously by Lindroos and co-workers [31].

For multipotency analysis, CELLstart pre-coating was used for well plates in all differentiation procedures except for chondrogenic differentiation. For osteogenic induction analysis, hASCs were seeded on 12-well plates at a density of 2.5 × 10^3 ^cells/cm^2 ^in HS MM. After 24 hours, the HS medium was replaced by OM3 in RegES medium (Table 1). The osteogenic differentiation was detected by alkaline phosphatase (ALP) staining at a 14-day time point. Briefly, cell cultures were fixed with 4% paraformaldehyde (PFA) and stained with a leukocyte ALP kit according to Sigma procedure 86 (Sigma-Aldrich, St Louis, MO, USA).

**Table 1:** The medium compositions used in the study.

Medium	Composition
Maintenance medium (MM)	10% FBS-, 10% HS-, or RegES-medium

OM1	100 nM Dex, 50 µM AsA2P, 10 mM β-GP in MM

OM2	10 nM Dex, 150 µM AsA2P, 10 mM β-GP in MM

OM3	5 nM Dex, 250 µM AsA2P, 10 mM β-GP in MM

Maintenance medium (MM) was culture medium with 10% FBS or 10% HS, or defined, xeno-free RegES-medium (composition defined in Additional file 1). The osteogenic media (OM) were supplemented with dexamethasone (Dex), L-ascorbic acid 2-phosphate (AsA2-P), and beta-glycerophosphate (β-GP). MM, maintenance medium; OM, osteogenic medium; FBS, fetal bovine serum; HS, human serum; Dex, dexamethasone; AsA2-P, L-ascorbic acid 2-phosphate; β-GP, beta-glycerophosphate.

For the adipogenic induction, hASCs were seeded on 12-well plates at a density of 2 × 10^4 ^cells/cm^2 ^in HS MM. After 2 days, RegES medium with adipogenic supplements was added: 33 µM biotin (Sigma-Aldrich), 1 µM Dex (Sigma-Aldrich), 100 nM insulin (Life Technologies), and 17 µM pantothenate (Fluka, Buchs, Switzerland). In addition to other adipogenic supplements, 250 µM isobutylmethylxanthine (IBMX; Sigma-Aldrich) was used for the first 24 hours of adipogenic induction. After 14 days of culture, the intracellular lipid accumulation was detected by Oil Red O staining. Cell cultures were fixed with 4% PFA, followed by treatment with 60% isopropanol, and stained with 0.5% Oil Red O solution (Sigma-Aldrich) in 60% isopropanol.

A micromass culture technique was used for the chondrogenic differentiation, where 1 × 10^5 ^cells were seeded on 24-well plates in a 10 µl volume, and allowed to adhere for 3 hours in +37°C prior to the addition of RegES medium with chondrogenic supplements: 1% ITS+1 (Sigma-Aldrich), 50 µM AsA2-P (Sigma-Aldrich), 55 µM sodium pyruvate (Life Technologies), 23 µM L-proline (Sigma-Aldrich), 10 ng/ml transforming growth factor-beta (Sigma-Aldrich). The chondrogenic differentiation was confirmed by histological Alcian Blue (Sigma-Aldrich) staining after 14 days of culture. The micromass pellets were fixed in 4% PFA, embedded in paraffin and sectioned at 5 µm thickness. Alcian Blue (pH 1.0) solution was used to detect sulphated glycosaminoglycans (GAGs) characteristic in cartilaginous matrices. Nuclear Fast Red (Biocare Medical, Concord, MA, USA) was used as a counterstain.

### Osteogenic medium compositions

For the comparison of the different OM compositions, hASCs were plated on 12-well plates at a density of 7 × 10^3 ^cells/cm^2^. The cells were plated in either HS or FBS MM and allowed to attach for 24 hours before starting the osteogenic differentiation. For xeno-free conditions, the well plates were pre-coated with CELLstart and hASCs were plated in HS MM for 24 hours to facilitate attachment of the cells before medium was replaced by RegES MM or RegES with osteogenic supplements (Table 1). Three different osteogenic medium compositions, OM1, OM2 and OM3, were compared in FBS, HS and xeno-free conditions (Table 1). Based on the literature, OM with lower Dex and higher AsA2-P concentration than in the traditionally used OM (OM1) was suggested to be more optimal for osteo-induction of hASCs in FBS-based medium [16]. We conducted a preliminary screening to test different concentrations of AsA2-P and Dex in HS-based medium (Additional file 2). Based on these results, OM2 and OM3 with low Dex and high AsA2-P concentrations were chosen for further comparison with the traditionally used OM1. AsA2-P, Dex and β-GP used in the osteogenic media were all purchased from Sigma-Aldrich. The hASCs cultured in FBS MM were used as a reference in all the analyses. It must be noted that RegES MM contains a high basal level of AsA2-P (50 µg/ml that corresponds to 170 µM). As a consequence, the additional AsA2-P in the osteogenic compositions raised the total concentrations of AsA2-P in RegES-based OM1, OM2 and OM3 to 220, 320, and 420 µM, respectively.

### Morphology and cell number

Cell number, based on the total amount of DNA per sample, was determined using a CyQUANT™ Cell Proliferation Assay Kit (CyQUANT; Molecular Probes, Invitrogen, Paisley, UK) as described previously [32]. Briefly, at 1-, 7- and 14-day time points the cells were lysed with 0.1% Triton-X 100 buffer (Sigma-Aldrich) and analyzed after a freeze-thaw cycle. Fluorescence was measured with a microplate reader (Victor 1420 Multilabel Counter; Wallac, Turku, Finland) at 480/520 nm. Morphology of the cells was observed at 1-, 3-, 7- and 14-day time points using light microscopy.

### Alkaline phosphatase activity and mineralization

The osteogenic differentiation capacity of hASCs was determined at 7 and 14 days by analyzing ALP activity and mineralization. ALP is a generally used marker for early osteogenic differentiation, whereas mineralization of the ECM is a characteristic of late osteogenic differentiation. The quantitative ALP analysis was performed on the same samples as the analysis of cell number, using the ALP Kit (Sigma-Aldrich) as reported previously [32]. A quantitative Alizarin Red S method was used at 7 and 14 days to detect mineralization as described previously [32]. Briefly, the ethanol fixed cells were stained with 2% Alizarin Red S solution (Sigma-Aldrich), and photographed after several steps of washing. Cetylpyridinium chloride (Sigma-Aldrich) was used to extract the dye, followed by absorbance measurement at 540 nm with a microplate reader (Victor 1420).

### Quantitative real-time PCR

Quantitative real-time reverse transcription polymerase chain reaction (qRT-PCR) analysis was used to compare the relative expression of osteogenic genes under different culturing conditions. For qRT-PCR analysis, hASCs were seeded on 6-well plates at a density of 7 × 10^3 ^cells/cm^2^. A CELLstart pre-coating of well plates was used in xeno-free conditions. Total RNA was isolated from the cells at 7- and 14-day time points with Nucleospin kit reagent (Macherey-Nagel GmbH & Co. KG, Düren, Germany) according to manufacturer's instructions. First-strand cDNA was synthesized from total RNA using the High-Capacity cDNA Reverse Transcriptase Kit (Applied Biosystems, Foster City, CA, USA). The expression of osteogenic genes including runx2A, DLX5, collagen type I, osteocalcin, and ALP was analyzed. Isoform A of runx2 was analyzed due to its specificity for osteogenic differentiation in comparison to isoform C [33,34]. Data was normalized to the expression of RPLP0 (human acidic ribosomal phosphoprotein P0), a housekeeping gene, which has shown to have stable expression under different experimental conditions in similar studies [35,36]. The primer sequences (Oligomer Oy, Helsinki, Finland) and accession numbers are presented in Table 2. The qRT-PCR mixture contained 50 ng cDNA, 300 nM forward and reverse primers, and SYBR Green PCR Master Mix (Applied Biosystems). The reactions were conducted with AbiPrism 7000 Sequence Detection System (Applied Biosystems) with initial enzyme activation at 95°C for 10 minutes, followed by 45 cycles of denaturation at 95°C for 15 seconds and anneal and extend at 60°C for 60 seconds. The expression levels of all differentiation cultures were compared to the expression level of FBS control cultures.

**Table 2:** The primer sequences for qRT-PCR.

Name		5'- Sequence -3'	Product size (bp)	Accession number
RUNX2A	Forward	CTTCATTCGCCTCACAAACAAC	62	NM_001024630.3
	
	Reverse	TCCTCCTGGAGAAAGTTTGCA		

DLX5	Forward	ACCATCCGTCTCAGGAATCG	75	NM_005221.5
	
	Reverse	CCCCCGTAGGGCTGTAGTAGT		

Collagen type I	Forward	CCAGAAGAACTGGTACATCAGCAA	94	NM_00088
	
	Reverse	CGCCATACTCGAACTGGAATC		

Osteocalcin	Forward	AGCAAAGGTGCAGCCTTTGT	63	NM_000711
	
	Reverse	GCGCCTGGGTCTCTTCACT		

Alkaline phosphatase	Forward	CCCCCGTGGCAACTCTATCT	73	NM_000478.4
	
	Reverse	GATGGCAGTGAAGGGCTTCTT		

RPLP0	Forward	AATCTCCAGGGGCACCAT T	70	NM_001002
	
	Reverse	CGCTGGCTCCCACTTTGT		

### Statistical analysis

Statistical analyses were performed with SPSS version 19 (IBM, Armonk, NY, USA). The effects of different culture conditions on cell number, normalized ALP activity, mineralization and relative gene expression were compared with nonparametric statistics using Kruskal-Wallis one-way analysis of variance by ranks, with Mann-Whitney post hoc test to analyze the specific sample pairs for significant differences. The significances obtained were corrected using Bonferroni adjustment in order to justify multiple comparisons. For example, the obtained *P *value was multiplied by the comparisons made within the time point (MM vs. OM1/OM2/OM3, OM1 vs. OM2/OM3, and OM2 vs. OM3 equals six comparisons within one time point), and multiplied with the number of time points (6 × 3 time points = 18, or 6 × 2 time points = 12). When for example *P *= 0.002 was obtained with Mann-Whitney, the *P *value was multiplied with 12 or 18, giving the final *P *values 0.024 or 0.036 respectively, depending the number of time points. The results were considered significant when *P *<0.05. The effect of culture duration on cell number was analyzed similarly using Kruskal-Wallis, Mann-Whitney and Bonferroni adjustment as described. All the results were standardized to the control condition (FBS MM). All the experiments were repeated three times using different donor in each repeat (n = 3). Technical triplicates of each sample were used in all the assays.

## Results

### Flow cytometric surface marker expression analysis

The flow cytometric analysis demonstrated that hASCs cultured in FBS and HS MM express the surface markers CD73, CD90, CD105 (Table 3). The hASCs lacked the expression of the CD14, CD19, HLA-DR, the hematopoietic marker CD45, and the vascular cell adhesion molecule CD106. The expression of CD34, CD49d and HLA-ABC was moderate. The results verified the mesenchymal origin of the hASCs, and the lack of hematopoietic and angiogenic markers [26,37,38].

**Table 3:** Characterization of hASCs.

Antigen	Surface protein	FBS	HS
CD14	Serum lipopolysaccharide-binding protein	2.6 ± 2.7	1.1 ± 1.0

CD19	B lymphocyte-lineage differentiation antigen	1.1 ± 0.9	0.4 ± 0.1

CD34	Sialomucin-like adhesion molecule	19.6 ± 12.2	27.4 ± 23.5

CD45	Leukocyte common antigen	1.5 ± 0.8	1.8 ± 1.6

CD49d	Integrin a2, VLA-4	12.7 ± 2.5	35.4 ± 10.1

CD73	Ecto-50-nucleotidase	82.2 ± 8.5	85.9 ± 7.3

CD90	Thy-1 (T cell surface glycoprotein)	97.3 ± 2.5	96.5 ± 4.6

CD105	SH-2, endoglin	85.3 ± 17.7	79.2 ± 21.1

CD106	VCAM-1 (vascular cell adhesion molecule)	0.6 ± 0.3	1.3 ± 1.7

HLA-ABC	Major histocompatibility class I antigens	16.9 ± 8.1	28.1 ± 16.4

HLA-DR	Major histocompatibility class II antigens	1.9 ± 1.2	1.0 ± 1.0

Surface marker expression of undifferentiated hASCs cultured either in FBS- or HS-medium as analyzed by flow cytometric analysis. Data is presented as mean ± standard deviation of the percentage of surface marker expression (n = 6). hASCs, human adipose stem cells; FBS, fetal bovine serum; HS, human serum; CD, cluster of differentiation.

### Analysis of multipotent differentiation capacity

**Figure 1:**
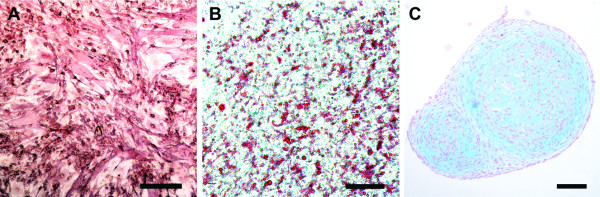
**Representative images showing the multilineage differentiation potential of hASCs cultured in RegES medium**. ALP staining for the osteogenic differentiation **(A)**, Oil Red O staining for the adipogenic differentiation **(B)**, and Alcian Blue staining for the chondrogenic differentiation **(C) **of hASCs. Scale bar 500 µm in (A), and 100 µm in (B) and (C). ALP, alkaline phosphatase; hASCs, human adipose stem cells.

The multipotent differentiation capacity of the hASCs cultured in RegES was examined by culturing the cells under osteogenic, adipogenic and chondrogenic conditions (Figure 1A-C). Differentiation was analyzed by histological stainings at a 14-day time point. Osteogenic differentiation was confirmed by a positive ALP staining at day 14 (Figure 1A). Adipogenic differentiation (Figure 1B) was verified by Oil Red O staining, showing the lipid droplets present in differentiated cells. Chondrogenic differentiation (Figure 1C) was confirmed by the formation of aggregates as well as Alcian Blue staining. The chondrogenically induced hASCs formed aggregates that were intensively stained by Alcian Blue, indicating the presence of sulphated GAGs typical to cartilage.

### Preliminary screening of the osteogenic medium compositions

Preliminary screening was conducted to test a hypothesis if OM with low Dex and high AsA2-P concentrations could be efficient osteo-inducer of hASCs in HS-based medium (Additional file 2). According to the preliminary screening, it was concluded that AsA2-P is needed for the effective osteogenic differentiation of hASCs, and higher concentrations of AsA2-P resulted in increased runx2 expression and ALP activity (data not shown). Dex was found to suppress proliferation and collagen type I expression at high concentration (100 nM), whereas low Dex concentration (5 nM) was beneficial for the osteogenic differentiation when combined with high AsA2-P concentration (250 µM). Consequently, the highest proliferation, ALP activity and runx2 expression were achieved with 150 µM AsA2-P and 10 nM Dex (OM2), and 250 µM AsA2-P and 5 nM Dex (OM3), compositions that were selected for further experiments to be compared with the traditionally used composition (OM1), containing 50 µM AsA2-P and 100 nM Dex.

### Morphology and cell number

**Figure 2:**
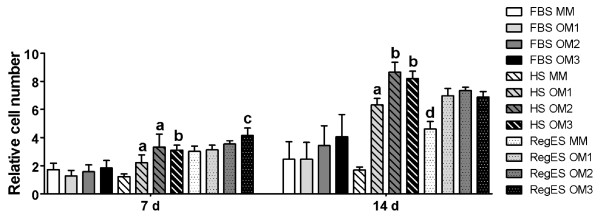
**Relative cell number**. Cell number at day 7 and 14 after culture in maintenance medium (MM) or different osteogenic media (OM1, OM2 or OM3) based on FBS, HS or RegES. Results are expressed as mean + SD. All results were standardized to the control condition (FBS MM at day 1). **(A) **(*P *<0.05) relative to HS MM; **(B) **(*P *<0.05) relative to HS MM and OM1; **(C) **(*P *<0.05) relative to RegES MM and OM1; **(D) **(*P *<0.05) relative to RegES OM1, OM2 and OM3, as well as HS MM and FBS MM. FBS, fetal bovine serum; HS, human serum; MM, maintenance medium; OM, osteogenic medium; SD, standard deviation.

**Figure 3:**
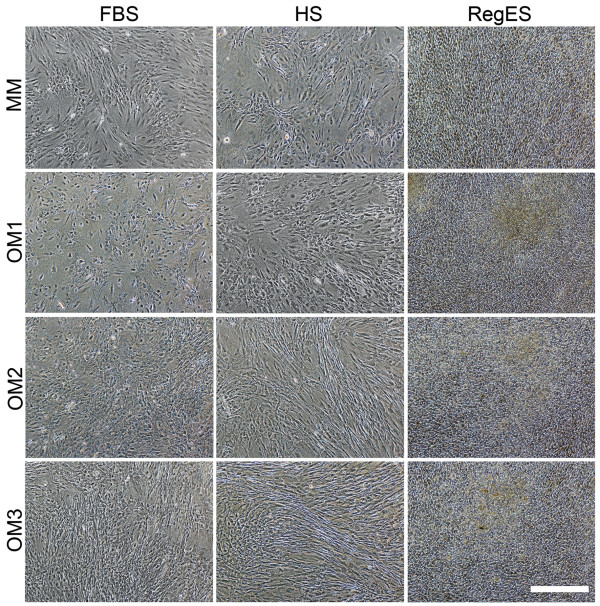
**Cell morphology in different media**. Representative light microscope images showing hASC morphology and proliferation at 14-day time point in each medium. Scale bar 500 µm. FBS, fetal bovine serum; hASCs, human adipose stem cells; HS, human serum; MM, maintenance medium; OM, osteogenic medium

Cell number was analyzed quantitatively at 7- and 14-day time points (Figure 2) along with observation of morphology (Figure 3 and Additional file 3). Cell number was also analyzed at day 1 to test for equal plating efficiency; this was confirmed by the similar cell numbers between FBS, HS and RegES conditions at day 1 (data not shown). All different maintenance media were able to support the proliferation of hASCs as the cell numbers increased significantly (*P *<0.05) with time when comparing day 1 and 14 within each serum condition. However, FBS MM and HS MM exhibited notably lower cell numbers than RegES MM (Figure 2). The increased growth rate of hASCs cultured in RegES MM was evident already after 7 days of culture, as was shown by cell number (Figure 2) and morphology analysis (Additional file 3). The cell number of RegES maintenance culture was significantly higher at day 14 (*P *<0.05) when compared to HS and FBS maintenance cultures.

All osteogenic media (OM1-3) increased cell number significantly when compared to the respective MM under HS and RegES conditions at day 14 (Figure 2). Although a similar trend could be detected in FBS medium at day 14 in Figure 2 and Figure 3, the difference between MM and OM compositions was not significant in the quantitative analysis. In contrast, the stimulating effect of OM in HS and RegES cultures was shown already at day 7; OM3 exhibited significantly higher cell number than OM1 and MM in both HS and RegES. At day 14, in HS cultures the cell number was significantly higher in OM2 and OM3 when compared to OM1 and MM. In RegES cultures all OM compositions resulted in higher cell number than MM at the 14-day time point, but there were no differences between OM1, OM2 or OM3. This may be due to the high growth rate of hASCs in RegES medium; the cells had already reached confluency by day 14 and grew in multiple layers (Figure 3).

### Alkaline phosphatase activity

**Figure 4:**
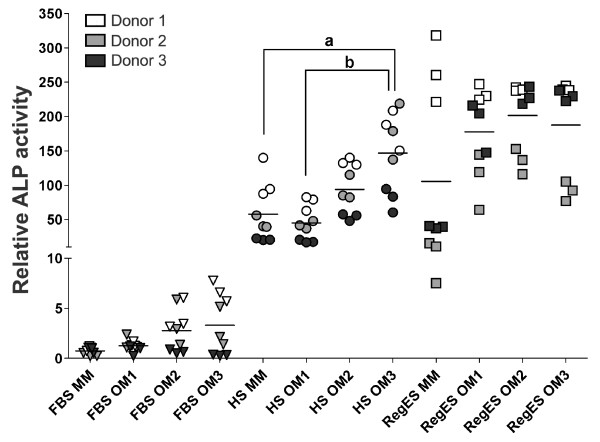
**Relative ALP activity on day 14**. The scatter plot shows mean ALP activity for each condition and individual data points for each donor. All results were standardized to the control condition (FBS MM at day 7). **(A) **(*P *<0.05) for the difference between HS OM3 and MM; **(B) **(*P *<0.01) for the difference between HS OM3 and OM1. ALP, alkaline phosphatase; FBS, fetal bovine serum; HS, human serum; MM, maintenance medium; OM, osteogenic medium.

The ALP activity of each sample was normalized to the amount of DNA in the sample, and all the results were considered relative to FBS MM at the 7-day time point. Due to notable donor variation, ALP activity is presented as a scatter plot showing the response of individual donors at day 14 (Figure 4). There were no statistically significant differences at day 7 (data not shown). At day 14, the ALP activity was notably low in all FBS cultures when compared to HS and RegES cultures. Two out of three donors exhibited a notable increase of ALP activity in response to OM2 and OM3 under FBS and HS conditions, whereas one donor did not seem to respond to any of the OM compositions. In RegES medium, the osteogenic supplements resulted in higher ALP activity with hASCs from two donors, whereas one donor line was induced by the RegES MM. Irrespective of the donor variation, the mean ALP activity of HS OM3 was significantly higher than with HS MM (*P *= 0.036) and OM1 (*P *= 0.006).

### Mineralization

**Figure 5:**
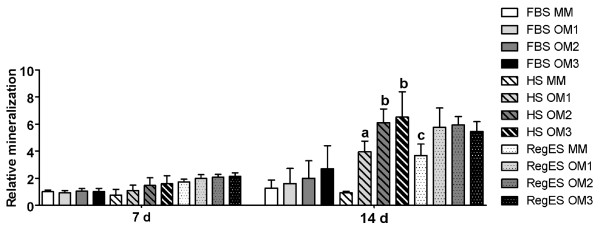
**Relative mineralization**. Quantitative mineralization of hASCs cultured in maintenance medium (MM) or different osteogenic media (OM1, OM2 or OM3) based on FBS, HS or RegES. Results are expressed as mean + SD. All results were standardized to the control condition (FBS MM at day 7). **(A) **(*P *<0.05) relative to HS MM; **(B) **(*P *<0.05) relative to HS MM and OM1; **(C) **(*P *<0.05) relative to RegES OM1, OM2 and OM3. FBS, fetal bovine serum; hASCs, human adipose stem cells; HS, human serum; MM, maintenance medium; OM, osteogenic medium; SD, standard deviation.

**Figure 6:**
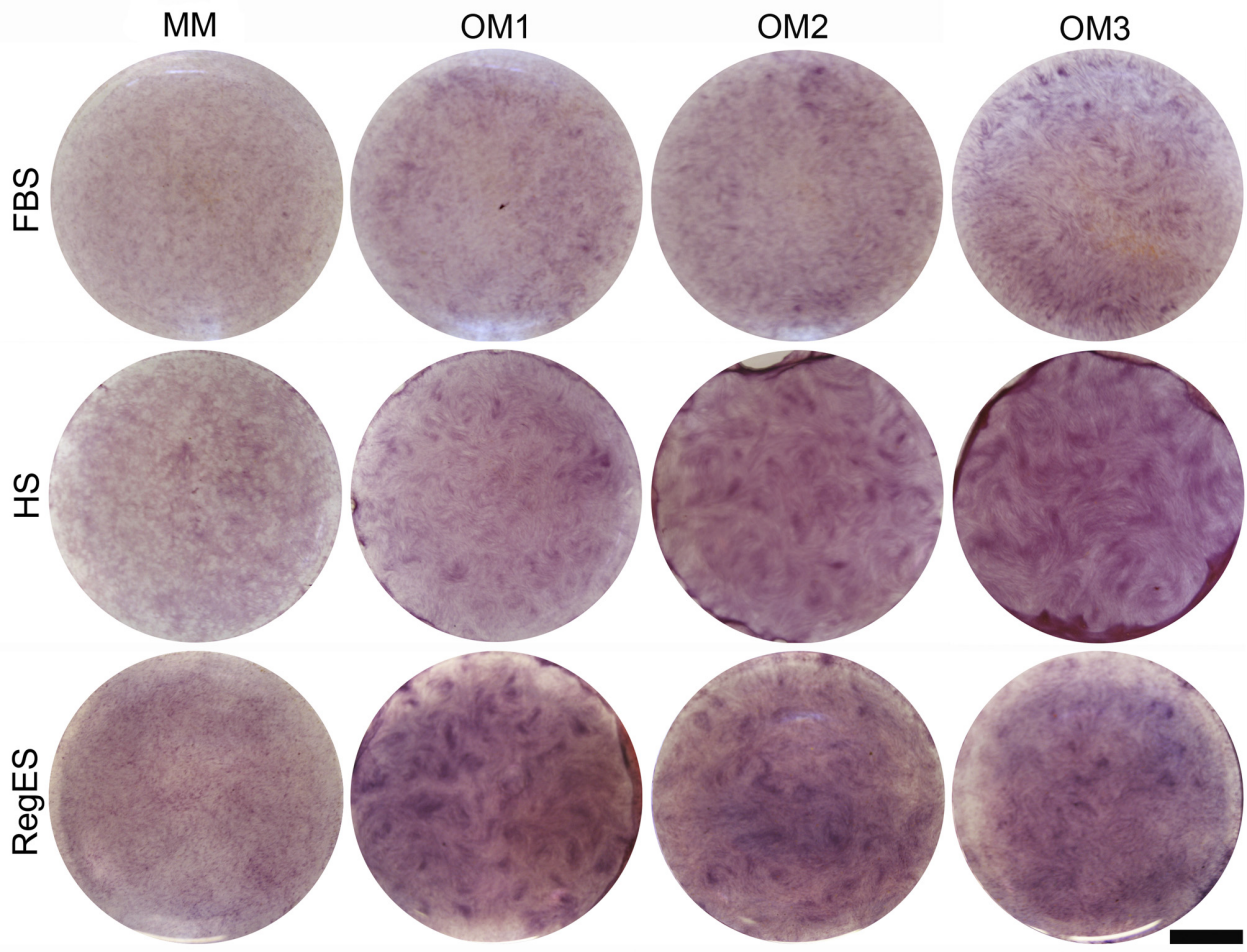
**Qualitative mineralization**. Representative images showing Alizarin Red staining to detect mineralization of hASCs cultured in maintenance medium (MM) or different osteogenic media (OM1, OM2 or OM3) based on FBS, HS or RegES at 14-day time point. Scale bar 500 µm. FBS, fetal bovine serum; hASCs, human adipose stem cells; HS, human serum; MM, maintenance medium; OM, osteogenic medium.

Similar to the ALP activity, OM3 increased mineralization the most in FBS cultures at day 14, but the difference to MM was not significant (Figure 5). Overall, mineralization was very weak under FBS conditions (Figures 5 and 6). In HS cultures, OM2 and OM3 resulted in a significantly higher mineralization than MM and OM1 at the 14-day time point. Although the difference between OM2 and OM3 in HS was not significant in the quantitative analysis of mineralization, the qualitative Alizarin Red staining showed more intense mineralization by OM3 than OM2 (Figure 6). In RegES cultures, all OM compositions induced significantly higher mineralization than RegES MM after 14 days of culture (Figures 5 and 6), but there were no significant differences between the different OM (Figure 5).

### Expression of osteogenic markers

**Figure 7:**
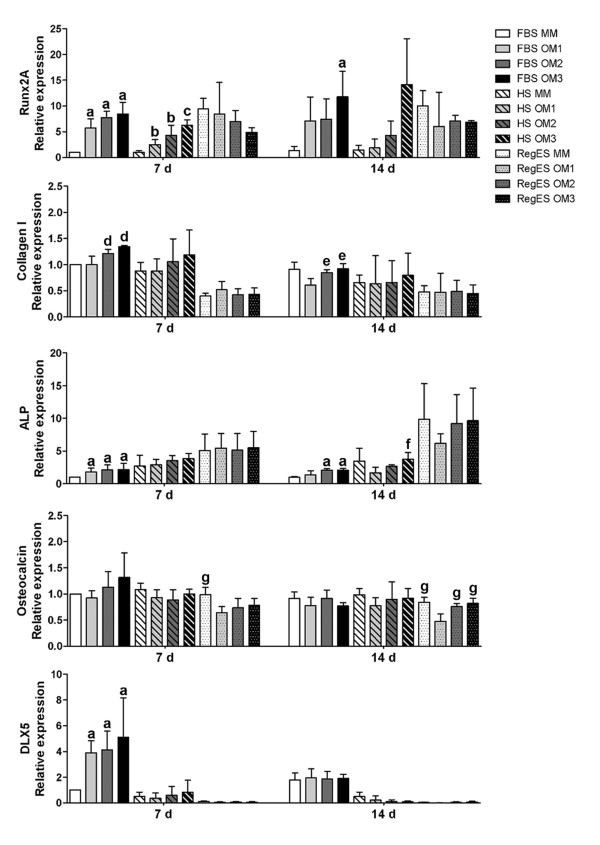
**Quantitative real-time PCR results**. The relative expression of osteogenic genes in hASCs cultured in maintenance medium (MM) or different osteogenic media (OM1, OM2 or OM3) based on FBS, HS or RegES at 7- and 14-day time points. Results are expressed as mean + SD. All results were standardized to the control condition (FBS MM at day 7). **(A) **(*P *<0.05) relative to FBS MM; **(B) **(*P *<0.05) relative to HS MM; **(C) **(*P *<0.05) relative to HS MM and OM1; **(D) **(*P *<0.05) relative to FBS MM and OM1; **(E) **(*P *<0.05) relative to FBS OM1; **(F) **(*P *<0.05) relative to HS OM1 and OM2; **(G) **(*P *<0.05) relative to RegES OM1. FBS, fetal bovine serum; hASCs, human adipose stem cells; HS, human serum; MM, maintenance medium; OM, osteogenic medium; SD, standard deviation.

Quantitative RT-PCR was used to detect relative changes in the expression of osteogenic marker genes runx2A, DLX5, collagen type I, osteocalcin, and ALP (Figure 7). The relative expression of runx2A, one of the key osteogenic transcription factors, was significantly induced by the osteogenic components in FBS and HS conditions. In FBS cultures, all OM compositions induced significantly higher expression of runx2A than MM at the 7-day time point. At day 14, FBS OM3 (*P *= 0.048) showed significantly higher expression of runx2A mRNA when compared to FBS MM. In HS cultures, all OM compositions exhibited significantly higher runx2A expression than HS MM (*P *= 0.024) at day 7, and OM3 upregulated the runx2A expression significantly when compared to OM1. At day 14, a high expression of runx2A was achieved by HS OM3, but due to donor variation there was no significant difference to HS MM. In RegES cultures, there were no statistically significant differences between the OM compositions and MM for the expression of runx2A, likely due to the high basal level of runx2 expression in RegES MM. The expression of DLX5, activator of runx2, was significantly upregulated by all OM compositions on day 7 under FBS conditions, when compared to FBS MM (*P *= 0.024). However, there were no significant differences on day 14. The level of DLX5 expression was low under HS and RegES conditions.

The expression of collagen type I, a major component of organic bone matrix, was significantly increased by OM2 and OM3 under FBS conditions, specifically at day 7 when compared to both MM and OM1. At day 14, the expression of collagen type I level was slightly lower, but yet the expression was significantly higher with OM2 and OM3 than OM1 (*P *= 0.024). There were no significant differences between the groups under HS and RegES conditions. The level of collagen type I mRNA was generally lower in RegES than in FBS or HS conditions.

In contrast to collagen, the expression of ALP was notably upregulated in RegES MM, OM2 and OM3 cultures when compared to FBS and HS cultures at the 14-day time point. Overall, the level of ALP expression was low in FBS medium. Nevertheless, significantly higher expression of ALP mRNA was detected in all FBS OM compositions at day 7, and by OM2 and OM3 at day 14, when compared to FBS MM (*P *= 0.024). In HS medium, OM3 showed significantly higher level of ALP expression at day 14 than OM1 (*P *= 0.024) and OM2 (*P *= 0.048), but not when compared to HS MM. Again there were no significant differences between RegES groups.

The expression of the late osteogenic marker osteocalcin was moderate in all of the cell cultures. Mild upregulation of osteocalcin expression by OM3 could be seen in FBS cultures at day 7, but the difference was not statistically significant. The elevated expression of osteocalcin in FBS OM3 was decreased to basal level by day 14. In RegES medium, the osteocalcin mRNA was downregulated by OM1, especially at the 14-day time point, where the level of osteocalcin in OM1 was significantly lower than in all the other RegES groups.

## Discussion

While the effects of the OM supplements have been largely studied with BMSCs [15,19-21], there are only limited studies with ASCs [16,22,39]. One of the major shortcomings in all of these *in vitro *studies has been the lack of comparison between different serum conditions. Due to quality and safety issues, the clinical hASC-based applications need to move from the animal-derived products to human-derived or more preferably to defined and xeno-free conditions [24]. However, most of the *in vitro *studies are still conducted using FBS. Given that the serum conditions can significantly affect the cell response, it is crucial to obtain research data with more clinical relevance [26,31].

Accordingly, we aimed to optimize the osteogenic culturing conditions for the *in vitro *induction of hASCs by testing different concentrations of Dex and AsA2-P in HS-based medium as well as in a defined, xeno-free medium RegES, with FBS medium functioning as a control. As hypothesized, the differential effect of FBS, HS or RegES media was evident in hASC proliferation and osteogenic differentiation. Comparison of the cell growth in FBS, HS and RegES maintenance media revealed a slightly higher growth rate in hASCs cultured in FBS MM than in HS MM, whereas the highest growth rate was achieved in RegES MM. When comparing MM and OM conditions in general, significantly higher cell number was achieved in OM than MM particularly in HS and RegES cultures. The effect of OM in cell number was also dependent of serum conditions; under FBS conditions OM3 with the highest AsA2-P concentration promoted cell growth most, but under HS conditions both OM2 and OM3 resulted in equally elevated cell numbers. High AsA2-P concentration (250 µM) has been reported to stimulate proliferation of BMSCs and osteoblast-like cells [21,40], whereas high Dex concentration (100 nM) may inhibit proliferation [22]. More importantly, high AsA2-P concentration may stimulate proliferation without a reciprocal loss of differentiation potency [21,41].

The osteogenic induction capacity of OM1, OM2 and OM3 was compared by analyzing the ALP activity, mineralization and relative expression of several osteogenic markers. Contrary to expectations, the level of osteogenic differentiation was the lowest in FBS-cultured hASCs as measured by ALP activity and mineralization. Another significant finding was that the RegES MM alone was found to induce the early osteogenic differentiation as shown by the elevated ALP activity, although supplementation with Dex, AsA2-P and β-GP was required to achieve mineralization. On the whole, the inductive effect of high AsA2-P and low Dex concentration, as in OM3, was most evident in HS cultures, resulting in high ALP activity and mineralization. Prior studies have noted the importance of AsA2-P in the osteogenic differentiation of BMSCs and osteoblastic cells [21,41-43]. In contrast, Dex in high concentrations has been shown to inhibit osteogenic differentiation [44,45], although it seems to be necessary for the efficient osteo-induction of MSCs in low concentrations [15,19,20,44].

In the present study, variation between donors could be detected particularly in ALP activity. Under HS and FBS conditions, two out of three donors exhibited a notable increase in ALP activity in response to OM2 and OM3, whereas one donor did not seem to respond to the OM supplements. Donor sample variability affects the interpretation of the results regardless of analyzing method and diminishes the statistical significance by increasing standard deviation. Others have established this problem previously with ASCs [11,23,46] and BMSCs [15,47,48]. With BMSCs, Jaiswal and co-workers showed that the basal level of ALP activity as well as the timing of the peak ALP activity varied greatly between the different donor samples irrespective of donor age [15]. The relative fold induction in ALP activity varied 1.5- to 6.4-fold depending on donor [15]. Similar kind of variation has been detected with ASCs as well [11,23,46].

The osteogenic effect of OM1, OM2 and OM3 under different serum conditions was further studied by analyzing the relative expression of osteogenic markers, runx2A, DLX5, collagen type I, osteocalcin and ALP. Overall, the qRT-PCR data demonstrated significant upregulation of runx2A mRNA under osteogenic differentiation in FBS and HS medium, and early stimulation of DLX5 under FBS conditions. The regulation of other markers, collagen type I, osteocalcin and ALP, was modest. When comparing the different OM compositions, OM2 and OM3 resulted in significantly higher expression of runx2A, collagen type I, and ALP, than corresponding MM under FBS or HS conditions. In some cases, OM2 and OM3 also resulted in higher expression of runx2A, collagen type I, and ALP than OM1. Under FBS conditions, OM3 induced significantly higher expression of runx2A when compared to FBS MM at day 14. In addition, collagen type I expression was significantly upregulated by FBS OM2 and OM3 at day 7 in comparison to both MM and OM1, and versus OM1 on day 14. Under HS conditions, OM3 resulted in significantly higher runx2A expression when compared to HS MM and OM1 at day 7. Moreover, HS OM3 resulted in higher level of ALP expression than OM1 and OM2 on day 14. Hence, greater expression of osteogenic markers can be achieved by OM with increased AsA2-P and lowered Dex, that is OM2 or OM3 composition.

In RegES medium, significant differences in the runx2A, collagen type I or ALP expression were not detected, likely due to the high basal level of expression in RegES MM. However, the level of osteocalcin mRNA was significantly lower in RegES OM1 than in MM, OM2 or OM3 at day 14. The expression of DLX5 appeared to peak already on day 7 under osteogenic induction under FBS conditions, whereas no regulation of DLX5 was detected under HS and RegES conditions.

There were also notable differences in the collagen type I and ALP mRNA expression between the serum conditions in general. The expression of collagen type I was notably lower in all RegES groups when compared to the respective FBS and HS groups. The expression of ALP, on the contrary, was higher in the RegES groups than in FBS and HS groups. This result correlates with the high ALP protein activity in RegES cultures, but in turn, not with the high ALP activity found in HS cultures. Unexpectedly, the expression of runx2, collagen type I, ALP or osteocalcin was not upregulated by time (day 7 versus 14) in any of the OM groups. Although we did not see significant upregulation by time, the culture period of 14 days has been shown to be sufficient for the detection of osteogenic gene expression [49-51]. Furthermore, considering clinical applications, the *in vitro *culture period should be minimized, as prolonged culture may increase the risk of contamination or genetic abnormalities. Therefore, the inductive effects *in vitro *should also appear within as short a time period as possible.

In contrast to ALP activity and mineralization, relatively small changes were detected in mRNA expression levels between MM and OM groups under all serum conditions. Although challenging the common conception, the lack of upregulation or even downregulation of certain osteogenic markers in ASCs upon osteogenic differentiation has been reported previously [12,18]. For OM-induced BMSCs, similar differences have been reported between the data obtained on protein level and real-time PCR for collagen type I expression [52]. The flow cytometric analysis showed significantly upregulated expression of collagen type I in the ECM of differentiated BMSCs, but there was no notable increase in collagen type I mRNA even after osteogenic differentiation according to real-time PCR [52]. While this phenomenon is still poorly understood, it is evident that the route of mRNA to protein is a highly regulated and complex pathway, where even small changes at transcriptional or posttranscriptional level can have a major phenotypic effect [12,18,53].

One of the main scopes of the present study was to investigate whether xeno-free RegES medium could be utilized for the efficient osteogenic differentiation of hASCs. Taken together, hASCs cultured in RegES showed increased osteogenic capacity, an effect likely explained by the composition of the RegES medium (Additional file 1). Apart from the several growth factors and cytokines, the high concentration of AsA2-P in RegES MM (50 µg/ml or 170 µM) may account for the high basal level of cell growth and osteogenic differentiation of hASCs in the plain RegES medium. Taking into account the additional AsA2-P in the osteogenic media, the concentrations in RegES OM1, OM2 and OM3, raised to 220, 320, and 420 µM, respectively. The maximal advantage in growth rate and differentiation in RegES-based osteogenic media was reached by AsA2-P concentrations varying from 220 to 320 µM, correlating to the results obtained with OM3 under FBS and HS conditions. However, as already mentioned, supplementation with Dex and β-GP in addition to AsA2-P was required for efficient osteo-induction and maturation of hASCs cultured in RegES.

## Conclusions

In summary, our results show that the serum conditions have a significant effect on the hASC behavior, such as proliferation and osteogenic differentiation capacity. Osteogenic differentiation of hASCs was the weakest in FBS-based medium. The plain RegES medium was able to induce early osteogenic differentiation of hASCs, although supplementation with Dex, AsA2-P and β-GP was needed to achieve mineralization. One of the key findings was that the commonly used OM1 supports the *in vitro *osteo-induction of hASCs poorly in FBS and HS medium. Instead, OM with higher AsA2-P and lower Dex should be used for the osteogenic differentiation of hASCs under FBS and HS conditions.

## Abbreviations

ALP: alkaline phosphatase; AsA2-P: L-ascorbic acid 2-phosphate; β-GP: beta-glycerophosphate; BMP: bone morphogenetic protein; BMSC: bone marrow-derived mesenchymal stem cell; Dex: dexamethasone; FBS: fetal bovine serum; GAGs: glycosaminoglycans; hASC: human adipose stem cell; HS: human serum; MM: maintenance medium; MSC: mesenchymal stem cell; OM: osteogenic medium; PFA: paraformaldehyde; qRT-PCR: quantitative real-time reverse transcription polymerase chain reaction.

## Competing interests

The authors KR and HS declare competing financial interests defined as patent application relating to the xeno-free culture medium RegES.

## Authors' contributions

LK carried out most of the cell culture studies and data analysis, drafted the manuscript, and participated in manuscript editing. SH designed and coordinated the study, had a major contribution in interpretation of data, and participated in writing and editing of the manuscript. BM carried out the flow cytometric analysis, participated in the data analysis and manuscript editing. HH carried out the statistical analysis and contributed to the interpretation of data. KR and HS conceived the RegES medium, and helped to draft and edit the manuscript. GS participated in the study design, coordination, and manuscript editing. SM participated in the study design and coordination, contributed to the interpretation of data, and participated in manuscript editing. All authors read and approved the final manuscript.

## Supplementary Material

Additional file 1 **RegES medium**. Complete formulation for RegES medium. Click here for file

Additional file 2 **Preliminary screening**. Osteogenic medium (OM) compositions of the preliminary screening (all in HS medium). Click here for file

Additional file 3 **Cell morphology at different time points**. Light microscope images showing representative hASC morphology in each maintenance medium at 3-, 7- and 14-day time points. Scale bar 500 µm. Click here for file
