# Supplementary figures and images for: Effects of different serum conditions on osteogenic differentiation of human adipose stem cells in vitro
Source: Stem Cell Res Ther. 2013 Feb 15;4(1):17. doi: 10.1186/scrt165 (PMC3706769; doi:10.1186/scrt165)

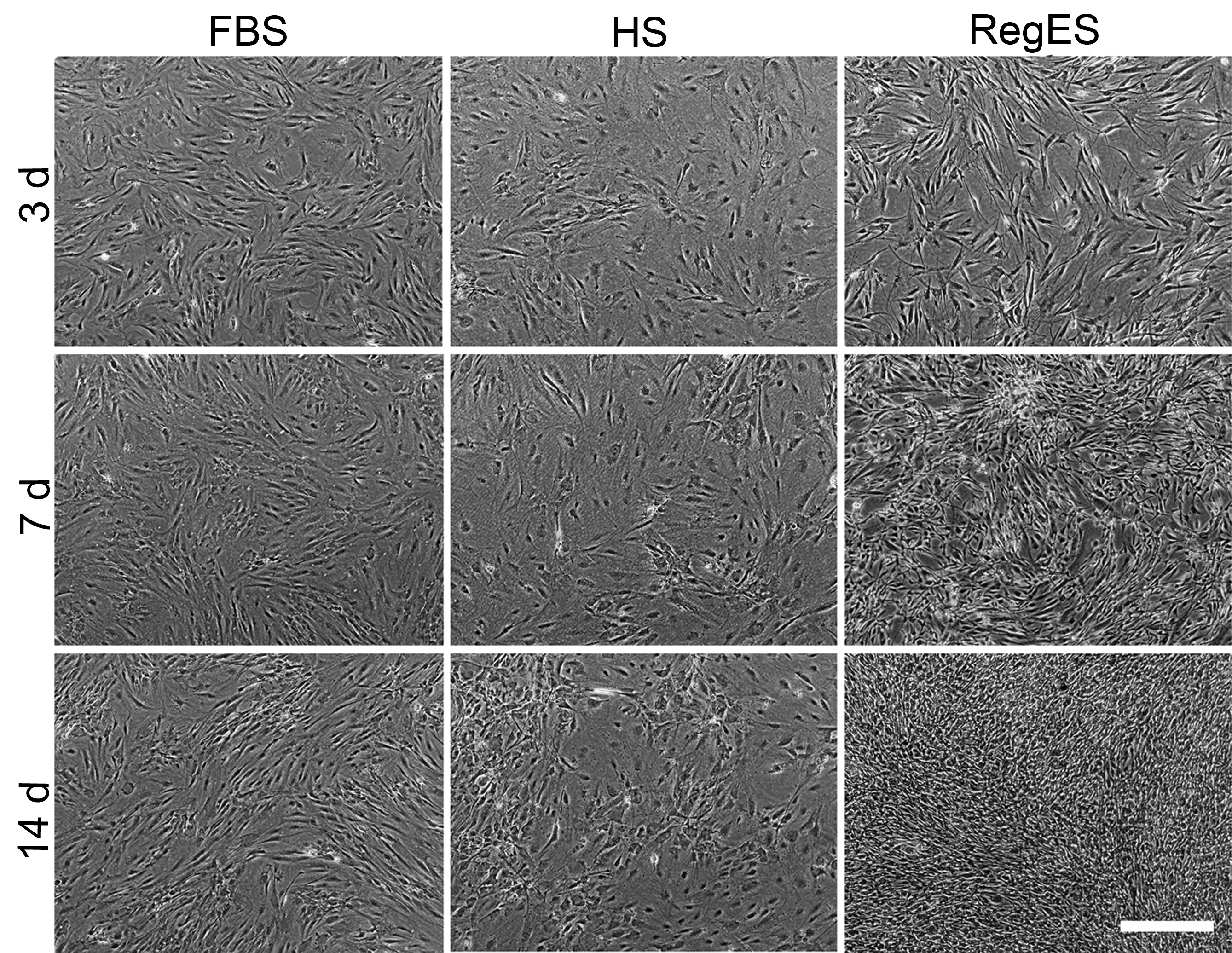

Supplement: Additional file 3 — Cell morphology at different time points. Light microscope images showing representative hASC morphology in each maintenance medium at 3-, 7- and 14-day time points. Scale bar 500 µm. [file scrt165-S3.TIFF]
